# Information about the US racial demographic shift triggers concerns about anti-White discrimination among the prospective White “minority”

**DOI:** 10.1371/journal.pone.0185389

**Published:** 2017-09-27

**Authors:** Maureen A. Craig, Jennifer A. Richeson

**Affiliations:** 1 Department of Psychology, New York University, New York, NY, United States of America; 2 Department of Psychology & Institution for Social and Policy Studies, Yale University, New Haven, CT, United States of America; 3 Department of Psychology & Institute for Policy Research, Northwestern University, Evanston, IL, United States of America; TNO, NETHERLANDS

## Abstract

The United States is undergoing a demographic shift in which White Americans are predicted to comprise less than 50% of the US population by mid-century. The present research examines how exposure to information about this racial shift affects perceptions of the extent to which different racial groups face discrimination. In four experiments, making the growing national racial diversity salient led White Americans to predict that Whites will face increasing discrimination in the future, compared with control information. Conversely, regardless of experimental condition, Whites estimated that discrimination against various racial minority groups will decline. Explorations of several psychological mechanisms potentially underlying the effect of the racial shift information on perceived anti-White discrimination suggested a mediating role of concerns about American culture fundamentally changing. Taken together, these findings suggest that reports about the changing national demographics enhance concerns among Whites that they will be the victims of racial discrimination in the future.

## Introduction

There’s a viewpoint that says, ‘I can fight for minorities, and I can fight for women,’ and if you get that, you make up a vast majority of the voting block and you win. And white males have been left aside a little bit in the politics of who speaks to them.

Sean Duffy (R-Wisconsin)

The morning after Donald Trump accepted the Republican Party’s nomination for President of the United States, Congressman Sean Duffy (R-Wisconsin) made the comments in the epigraph during a CNN interview [1], implying that White men have found someone who will speak to and for them in Trump. Just a few hours later, former Ku Klux Klan leader David Duke announced his intention to run for the United States Senate, declaring “what makes me different is I also demand respect for the rights and the heritage of European Americans” [2]. These expressions of concern regarding the treatment of Whites in contemporary American politics and broader society are echoed by several recent high-profile Supreme Court cases in which White plaintiffs have alleged that they were the victims of anti-White racial discrimination [3], [4]. For example, in both 2013 and 2016, Abigail Fisher attempted (unsuccessfully) to convince the Court that the University of Texas’ admissions process discriminated against her because she is White. In 2009, however, a group of primarily White firefighters won their case alleging that they were racially discriminated against when the New Haven fire department discounted test results that were to be used for promotion decisions, ironically, a decision that was made out of concern that utilizing the results would be discriminatory against Black firefighters [4].

While these examples highlight the apparent growth in claims of anti-White racial disregard and discrimination, another recent Supreme Court case reveals some Justices’ perceptions that racial minorities face ever decreasing levels of discrimination in American society. Specifically, in *Shelby County v*. *Holder* (2013) [5], key aspects of the 1965 Voting Rights Act, a law intended to reduce discrimination against minority voters, were struck down. In the majority opinion, Chief Justice Roberts argued that “our country has changed, and while any racial discrimination in voting is too much, Congress must ensure that the legislation it passes to remedy that problem speaks to current conditions.”

Taken together, these examples point to a seemingly broader zeitgeist wherein many White Americans believe that anti-White discrimination is on the rise, whereas the discrimination faced by racial minorities is rapidly decreasing. Indeed, in a recent survey, White respondents reported that anti-Black bias has sharply declined from the 1950s to the 2000s while anti-White bias has increased [6]. White participants, on average, reported that anti-White bias in the 2000s was even more prevalent than anti-Black bias [6]. Building on this work, the present research sought to examine White Americans’ perceptions of the amount of discrimination faced by Whites and racial minorities currently as well as their expectations regarding the discrimination these groups will face in the future. Further, the present work considers whether these seemingly increasing claims and/or expectations of anti-White discrimination might be due, at least in part, to rising societal racial diversity.

### Racial diversity and threat

One interesting and, perhaps, underappreciated feature of the aforementioned *Shelby County v*. *Holder* (2013) [5] case is that Shelby County recently experienced a tremendous increase in its racial diversity. Specifically, Shelby County’s Hispanic population grew over 2000% between 1990–2011 [7]. This type of growth is, of course, consistent with national trends, such that non-Hispanic Whites are expected to be less than 50% of the United States population by mid-century [8]. Recent research from several social scientific fields has explored the implications of this demographic trend for a number of different outcomes. Specifically, making increasing societal racial diversity salient leads members of the current majority group (Whites) to perceive that their group’s status (e.g., societal status and status as the prototypical American) is under threat, which in turn, affects both individuals’ racial attitudes and support for a variety of political issues [9–14]. For example, making growing national racial diversity salient leads Whites to express greater levels of anger towards and fear of racial minority groups [13], express more negative attitudes regarding racial minorities [9, 14], endorse more politically conservative positions on race-related (and race-neutral) policies [10, 12, 15], and express less support for cultural diversity [11].

The racial composition of individuals’ neighborhoods also predicts similar attitudinal and ideological patterns. For example, Whites who live in areas with a greater proportion of Blacks are also more likely to be registered as and vote Republican [16–17], and tend to express greater levels of bias against racial minorities [18–20]. Taken together, this work from sociology, social psychology, and political science suggests that increasing racial diversity may be threatening to White Americans, eliciting a number of important intergroup and political outcomes.

### Racial diversity and perceptions of anti-White discrimination

The primary aim of the present research was to examine whether increasing racial diversity may also elicit concerns about anti-White discrimination among White Americans, particularly in a future “majority-minority” United States. Relatively little empirical research has examined Whites’ perceptions of anti-White discrimination (for notable exceptions, see [6, 21–23]). Research examining the conditions under which Whites perceive discrimination against their group, however, suggests that one trigger of perceived anti-White racial bias is the perception that minorities are making considerable societal progress (e.g., in educational attainment, elected office), especially among Whites who endorse beliefs that justify the current racial status hierarchy [23–24]. Particularly relevant to the present work, research finds that organizational messages that are favorable to racial diversity lead Whites to perceive that they are likely to face more discrimination personally and that Whites as a group are also likely to face more discrimination, compared with neutral organizational messages [21]. This work, considered in tandem with the research reviewed previously suggesting that making increasing racial diversity salient can activate concerns that the status of minorities is rising relative to that of Whites, suggests that making the increasing racial diversity of the nation salient may also increase Whites’ perceptions that their racial group will face discrimination.

A secondary aim of the present research was to experimentally explore several potential psychological mechanisms through which salient national racial demographic shift information may influence White Americans’ perceptions of anti-White discrimination. Specifically, the influence of information about growing diversity on Whites’ intergroup attitudes and political ideology has been found to stem from perceived threats to Whites’ resources or status (e.g., [9–10, 13]) and perceived threats regarding Whites’ place as prototypical Americans [11]. We examine whether these threats may also account for any observed effects of the salient racial shift information on Whites’ perceptions of anti-White discrimination. Further, given that perceptions that institutions favor racial diversity can lead Whites to perceive more anti-White discrimination (e.g., [21]), we also test whether this mechanism (i.e., concerns about race-conscious decision-making) may similarly underlie perceptions of anti-White discrimination in the present research.

### Overview of the present research

Four experiments test whether making the growing racial diversity of the US salient influences White Americans’ perceptions of discrimination currently faced by Whites and racial minorities as well as their projections for future levels of discrimination. As outlined previously, we predict that Whites for whom the growing racial diversity of the nation is made salient will express greater concern about future anti-White discrimination compared with Whites for whom control information is made salient. Consistent with prior research [6], perceptions of anti-racial minority discrimination, by contrast, are expected to decline between the present and future. Finally, across studies, we test several potential reasons why information about increasing diversity may influence Whites’ perceptions of discrimination (e.g., concerns about group status; concerns that American society will change).

### Ethics information

The present research was conducted under the approval of the Institutional Review Board (IRB) at the Ohio State University (the first author’s former affiliation). In the laboratory studies (Studies 1, 2, and 4), an experimenter read an IRB-approved consent script to participants who indicated their consent to participate verbally before completing any study materials. In all studies, the first screen of the study was an IRB-approved study information page and participants were instructed to proceed to the study only if they consented to participate.

## Study 1

Study 1 provides an initial test of the effect of exposure to information about increasing national racial diversity on White Americans’ level of concern about anti-White discrimination. Specifically, White participants read information about the growing racial/ethnic diversity of the nation, including the so-called “majority-minority” shift (US racial shift condition), or they read control information [9–10] prior to indicating their expectations regarding the current and future prevalence of racial discrimination toward a variety of racial groups, including White Americans, as well as their political attitudes.

Further, to examine experimentally whether concerns about group status are the reason why exposure to information about the increasing national racial diversity may lead Whites to expect greater anti-White discrimination in the future, in addition to the US racial shift and control conditions, Study 1 also included an experimental condition found in previous work (i.e., [10]) to assuage Whites’ perceived group status threat in a future, far more racially diverse, nation. Specifically, after reading about the projected racial demographic shift in the nation, participants in this *assuaged threat* condition read that status relations in the US will largely remain the same as current relations. Participants then reported on their perceptions that different racial groups (i.e., White Americans, Hispanics/Latinos, Black Americans, Asian Americans, Native Americans) currently face discrimination and will face discrimination in the future. After, they reported their attitudes regarding a number of political issues.

Consistent with past work [10], we predicted that making the US racial population shift salient would elicit more support for conservative policies, but also, greater concern about anti-White discrimination in the future, compared with exposure to the control information. Further, we tested whether exposure to information that Whites’ societal status would remain high despite the racial shift would reduce any observed effects that the racial shift information alone had on perceptions of discrimination, suggesting that the racial shift effects were due to group status threat (as has been previously documented with political attitude shifts; [10]).

### Method

#### Participants

One hundred and seventy-nine White undergraduates (11 women, 168 men, *M*_age_ = 19.27, *SD*_age_ = 3.17, median family household income reported as between $100,000 and $119,999) at Ohio State University took part in the experiment in exchange for partial course credit. A gender imbalance is present for Study 1 (and Study 2), because for most of data collection, female participants were filtered into another study immediately following the initial demographic questions. Data collection spanned September to December, 2014.

#### Procedure and materials

Participants provided informed consent and completed an initial set of questions in which they indicated their demographic group memberships (e.g., race, gender) and a baseline measure of their political ideology. To assess baseline political ideology, among the initial demographics questions, participants also indicated their agreement with conservative and liberal political ideology (“I endorse many aspects of [conservative/liberal] political ideology” with 1 = *Strongly disagree*, 7 = *Strongly agree*). Due to a high correlation between the two items, *r* = –.75, *p* < .001, the liberal endorsement item was reverse-scored and the items were averaged to create a single index of baseline endorsement of conservative political ideology (*M* = 4.24, 95% CI[4.02, 4.46], *SD* = 1.50).

To manipulate exposure to race-related or race-neutral demographic change information, we utilized a newspaper article paradigm that has been used in prior research (see [10], Study 3). Participants were instructed that they would read articles and answer questions on social and political topics. All participants read and answered comprehension questions about three newspaper articles. The first two articles were the same across conditions (an article on a lawsuit against McDonald’s and an op-ed about plagiarism). The final article either reported on a) the growth of the rate of geographic mobility in the US (*control* condition), b) the projected racially diverse future US racial demographics (*US racial shift* condition), or c) the projected racially diverse future US racial demographics article with an additional paragraph designed to assuage racial status threat (*assuaged threat* condition). Specifically, the additional paragraph stated that despite the increased numerical representation of racial minority groups, status relations in the US will remain the same (i.e., White Americans will have higher incomes and wealth compared to other racial groups; see S1 Appendix for full text). To check that participants understood the information from the experimental manipulation, after reading the article, participants responded to questions intended to assess their comprehension of the target article (e.g., “Which racial group is expected to be the largest contributor to the population growth in the US?”).

Participants then indicated their perception that different racial groups (i.e., *White Americans*, *Hispanics/Latinos*, *Black Americans*, *Asian Americans*, *Native Americans*) a) are currently facing discrimination and b) will face discrimination in the future (1 = *Not at all*, 10 = *Very much*; [6]).

Next, participants indicated their positions on different political issues. Specifically, participants were asked about their support for: increasing/decreasing the required time to be eligible for US citizenship, affirmative action, increasing/decreasing foreign immigration to the US, establishing English as the official US language, drilling in the Arctic National Wildlife Refuge, utilizing “enhanced interrogation” techniques, increasing/decreasing the minimum wage, and support for universal health care. Items asking about increasing/decreasing policies were anchored by 1 = *Increased a lot* and 5 = *Decreased a lot* (the minimum wage item final response option was 6 *= Eliminate the minimum wage completely*). Policy support items were anchored by 1 = *Strongly opposed* and 7 = *Strongly in favor*. Because anchors differed across items, responses were standardized. We created an index of overall conservative policy endorsement (all 8 items; α = .72) with higher numbers indicating more conservative policy positions [25–26]. Finally, all participants completed additional demographic questions (e.g., age) and were debriefed.

### Results

Three participants who incorrectly responded to the items assessing comprehension of the target article were removed from analyses (the significance and direction of the results do not differ if these data are included). Thus, the final sample included 176 participants (60 control condition, 56 US racial shift condition, 60 assuaged threat condition).

#### Political attitudes

We first tested whether the previously-documented effect of information about the racial shift towards increased diversity on policy attitudes [10] was replicated in the present data. An ANCOVA examining the effect of experimental condition on policy attitudes, controlling for baseline political ideology, revealed a main effect of experimental condition, *F*(2, 172) = 3.80, *p =* .024, η_p_^2^ = 0.04. Replicating prior work, participants informed of the US racial demographic shift (*M*_adjusted_ = 0.13, 95% CI[0.02, 0.25], *SE* = 0.06) supported conservative positions more than did participants informed of geographic mobility (*M*_adjusted_ = -0.08, 95% CI[-0.19, 0.03], *SE* = 0.06), *F*(1, 172) = 6.64, *p =* .011, η_p_^2^ = 0.04. And, participants informed of the US racial demographic shift alone supported conservative positions more than did participants informed that Whites would maintain their (high) status in the “majority-minority” society (*M*_adjusted_ = -0.05, 95% CI[-0.16, 0.07], *SE* = 0.06), *F*(1, 172) = 4.68, *p =* .032, η_p_^2^ = 0.03. Participants informed that Whites would maintain their social status in the racially-diverse “majority-minority” nation did not express significantly more conservative positions than did control participants, *F*(1, 172) < 1, *p =* .674. This replicates prior research [10], and suggests that the assuaged threat information reduced concerns about Whites’ societal status, which, in turn, eliminated the shift towards conservatism otherwise observed due to information about the racial demographic changes.

#### Perceived anti-White discrimination

**Fig 1:**
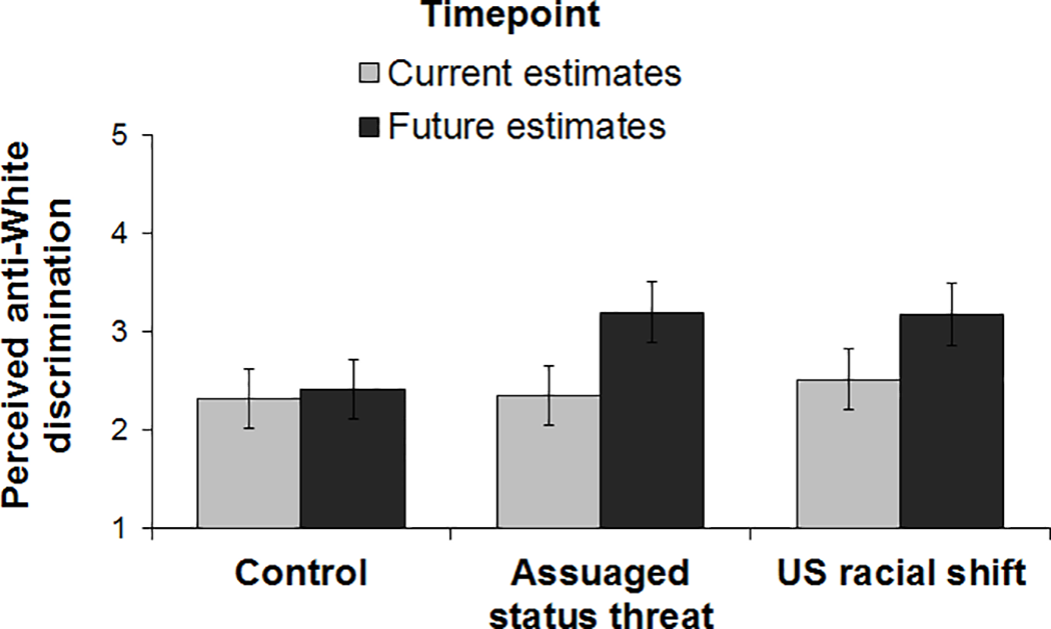
Perceived anti-White discrimination by experimental condition and timepoint (Study 1). Error bars represent within-subjects 95% confidence intervals [27].

A 3(condition: control, US racial shift, assuaged status threat) x 2(timepoint: current estimates, future estimates) mixed-design ANOVA on perceived anti-White discrimination revealed a main effect of timepoint, *F*(1, 173) = 17.55, *p* < .001, η_p_^2^ = 0.09, qualified by the experimental condition x timepoint interaction, *F*(2, 173) = 3.15, *p =* .045, η_p_^2^ = 0.04. As shown in Fig 1, participants in the control condition did *not* expect more anti-White discrimination in the future compared with current levels, *F*(1, 173) < 1, *p* = .649. Consistent with predictions, however, participants in the US racial shift condition expected more anti-White discrimination in the future compared with current levels, *F*(1, 173) = 8.47, *p* = .004, η_p_^2^ = 0.05. Somewhat surprisingly, participants in the assuaged status threat condition also expected more anti-White discrimination in the future compared with current levels, *F*(1, 173) = 15.01, *p* < .001, η_p_^2^ = 0.08, suggesting that concerns about group status are *not* the cause of the effects of the racial shift information on perceptions of anti-White discrimination. Further, a contrast test (contrast codes: 2–1–1) revealed that, controlling for perceptions of current anti-White discrimination, participants in the control condition estimated less future anti-White discrimination than participants in either of the conditions in which the racial shift was salient, *F*(1, 172) = 5.93, *p* = .016, η_p_^2^ = 0.03.

#### Perceived anti-racial minority discrimination

In contrast to the results for anti-White discrimination, analyses of the estimates of perceived discrimination against the different racial minority groups revealed only a main effect of timepoint [Blacks: *F*(1, 173) = 109.19, *p* < .001, η_p_^2^ = 0.39; Latinos: *F*(1, 173) = 58.65, *p* < .001, η_p_^2^ = 0.25; Asian Americans: *F*(1, 171) = 31.91, *p* < .001, η_p_^2^ = 0.16; Native Americans: *F*(1, 173) = 54.10, *p* < .001, η_p_^2^ = 0.24]. Participants expected that racial minority groups would face less discrimination in the future, compared to current levels, regardless of the experimental condition to which they were assigned (see Table 1 for descriptive statistics).

**Table 1:** Studies 1–4: Descriptive statistics for perceived discrimination by experimental condition and timepoint.

		Current estimates of discrimination					Future estimates of discrimination			
	Control condition	Racial shift condition	Assuaged threat condition	Colorblind-future condition	Assimilation-future condition	Control condition	Racial shift condition	Assuaged threat condition	Colorblind-future condition	Assimilation-future condition
*Targets of discrimination*	*M* (*SD*)	*M* (*SD*)	*M* (*SD*)	*M* (*SD*)	*M* (*SD*)	*M* (*SD*)	*M* (*SD*)	*M* (*SD*)	*M* (*SD*)	*M* (*SD*)
White Americans										
Study 1	2.32 (1.83)	2.52 (2.23)	2.35 (1.58)	–	–	2.42 (2.20)	3.18 (2.72)	3.20 (2.53)	–	–
Study 2	2.25 (2.01)	2.31 (1.86)	2.21 (1.98)	–	–	2.23 (2.07)	2.84 (2.17)	2.83 (2.45)	–	–
Study 3	3.38 (2.63)	3.32 (2.52)	–	3.33 (2.48)	–	3.57 (2.83)	4.04 (2.68)	–	3.91 (2.75)	–
Study 4	2.48 (1.81)	2.06 (1.71)	–	–	2.14 (1.68)	2.23 (1.69)	2.48 (2.28)	–	–	2.10 (1.68)
Black Americans										
Study 1	5.92 (2.23)	5.89 (2.86)	5.75 (2.16)	–	–	4.27 (2.27)	4.46 (2.64)	4.22 (2.29)	–	–
Study 2	6.45 (2.20)	6.32 (2.28)	6.30 (2.28)	–	–	4.95 (2.57)	4.51 (2.36)	4.25 (2.20)	–	–
Study 3	6.63 (2.34)	7.31 (2.00)	–	6.27 (2.26)	–	5.70 (2.63)	6.00 (2.21)	–	4.64 (2.40)	–
Study 4	6.73 (1.99)	6.65 (2.12)	–	–	6.36 (2.34)	5.27 (2.33)	5.04 (2.44)	–	–	4.60 (2.56)
Hispanics/Latinos										
Study 1	5.92 (1.97)	5.29 (2.28)	5.80 (1.93)	–	–	4.83 (2.22)	4.00 (2.26)	4.63 (2.40)	–	–
Study 2	5.95 (1.94)	5.70 (1.96)	5.70 (2.20)	–	–	4.61 (2.26)	4.08 (2.24)	4.09 (2.28)	–	–
Study 3	5.83 (2.36)	6.24 (2.04)	–	5.77 (2.16)	–	5.22 (2.64)	5.12 (2.20)	–	4.13 (2.31)	–
Study 4	6.08 (2.07)	6.40 (1.93)	–	–	6.00 (2.46)	5.08 (2.43)	5.42 (2.30)	–	–	4.44 (2.41)
Native Americans										
Study 1	4.17 (2.42)	4.45 (2.53)	4.35 (2.09)	–	–	3.22 (2.17)	3.52 (2.02)	3.55 (2.17)	–	–
Study 2	4.00 (2.15)	4.05 (2.11)	4.12 (2.06)	–	–	3.15 (2.09)	3.15 (1.87)	3.16 (2.05)	–	–
Study 3	5.36 (2.49)	5.89 (2.32)	–	5.08 (2.44)	–	4.51 (2.56)	5.10 (2.30)	–	4.09 (2.44)	–
Study 4	4.71 (2.29)	4.90 (2.38)	–	–	4.52 (2.79)	3.62 (2.14)	3.96 (2.47)	–	–	3.16 (2.37)
Asian Americans										
Study 1	4.53 (2.04)	4.34 (2.02)	4.31 (1.99)	–	–	3.49 (1.96)	3.73 (2.23)	3.78 (2.02)	–	–
Study 2	3.97 (1.56)	4.15 (1.91)	4.24 (1.83)	–	–	2.96 (1.56)	3.27 (1.80)	3.34 (1.92)	–	–
Study 3	4.43 (2.32)	5.23 (1.99)	–	4.47 (2.33)	–	3.91 (2.40)	4.55 (2.08)	–	3.66 (2.05)	–
Study 4	4.06 (1.66)	4.52 (2.17)	–	–	3.92 (1.96)	3.31 (1.79)	3.52 (2.17)	–	–	2.82 (1.71)

### Discussion

Overall, Study 1 replicated and extended past research [10, 12] finding that exposure to information about the rapidly diversifying racial composition of the nation leads White participants to express greater support for conservative policies and anticipate experiencing more anti-White discrimination in the future, compared with making geographic mobility (control information) salient. Perceptions of discrimination faced by racial minority groups, however, were not affected by the experimental manipulation; rather, Whites expected racial minorities to face less discrimination in the future compared with the present, regardless of their experimental condition.

Whereas assuaging group status threat by suggesting that Whites will remain in a high-status position in a future “majority-minority” nation eliminated the shift towards conservative political positions, participants’ expectations that anti-White discrimination will increase in the future were not alleviated by this assurance. These results suggest that the assuaged threat information successfully mitigated concerns about Whites’ social status in the present samples. Further, while concerns about group status or resources may drive the political shift in response to the changing racial demographics information, group status concerns do not appear to account for the effects of the racial shift information on perceptions of anti-White discrimination.

## Study 2

The results of Study 1 provide evidence that Whites’ perceptions of increasing anti-White discrimination upon exposure to the shifting demographics of the nation are unlikely attributable to concerns about Whites’ relative material status in society. To provide insight into what may drive Whites’ concerns about increasing anti-White discrimination, in Study 2 we sought to examine more closely which domains or types of discrimination that White participants may expect to increase in a more racially diverse United States. As in Study 1, White participants read about the US racial shift alone, the US racial shift accompanied by information known to assuage group status threat, or control information, then reported on their perceptions that different racial groups (i.e., White Americans, Hispanics/Latinos, Black Americans, Asian Americans, Native Americans) currently face discrimination and will face discrimination in the future. In addition, participants reported on the extent to which Whites will face discrimination in the future in a number of specific domains (e.g., political influence, interpersonal interactions, college admission, hiring). Based on the results of Study 1, we predicted that Whites who read about the racial shift, with or without the status-threat assuaging information, would expect greater anti-White discrimination in the future compared to the present, and when asked to specify the domains in which they expected greater future discrimination, they would indicate domains most relevant to material wealth (employment) as well as perhaps more interpersonal and cultural domains. Participants in the control condition were not expected to reveal greater expectations about anti-White discrimination in the future compared with the present.

### Method

#### Participants

Two hundred and twenty-eight White participants (48 women, 177 men, 3 did not specify gender, *M*_age_ = 18.98, *SD*_age_ = 1.50, median family household income reported as between $100,000 and $119,999) at Ohio State University took part in the experiment for partial course credit. Data collection spanned September to December, 2015.

#### Procedure and materials

Participants provided informed consent and completed an initial set of demographic questions; then the same newspaper article paradigm described in Study 1 provided the experimental manipulation (control condition, US racial shift condition, assuaged threat condition). Next, participants completed the same perceived discrimination items as in Study 1. After, in order to get a better sense of the domains in which Whites anticipate facing discrimination, participants were also asked to indicate (1 = *Not at all*, 10 = *Very much*) how much Whites will face discrimination in the future in a number of domains (e.g., political influence, interpersonal interactions, college admission, hiring decisions; see Table 2 for the list of domains). Finally, as in Study 1, participants completed additional demographic questions and were debriefed.

**Table 2:** Factor loadings (exploratory factor analysis) for domains of anti-White discrimination (Study 2).

	*Employment & Education*	*Interpersonal*
Scholarships	**.946**	-.021
College admission	**.871**	.009
Hiring decisions	**.402**	.389
Interpersonal interactions	-.014	**.858**
Interactions with police	-.110	**.840**
Housing	-.028	**.785**
Dating	.016	**.736**
Free speech	.054	**.724**
Expressing their culture/traditions	.009	**.707**
Political influence	.166	**.673**

*Note*. This exploratory factor analysis used the principal axis method of factor extraction with oblique rotation.

### Results

Two participants who incorrectly responded to the target article comprehension questions were removed from analyses (the significance and direction of results do not differ if these individuals are included, with one exception: the experimental condition x timepoint interaction for the general perceived anti-White discrimination analysis was marginally significant, *F*(2, 224) = 3.02, *p* = .051, η_p_^2^ = 0.03). Thus, the final sample included 226 participants (75 US racial shift condition, 76 assuaged threat condition, 75 control condition).

#### Perceived anti-White discrimination

**Fig 2:**
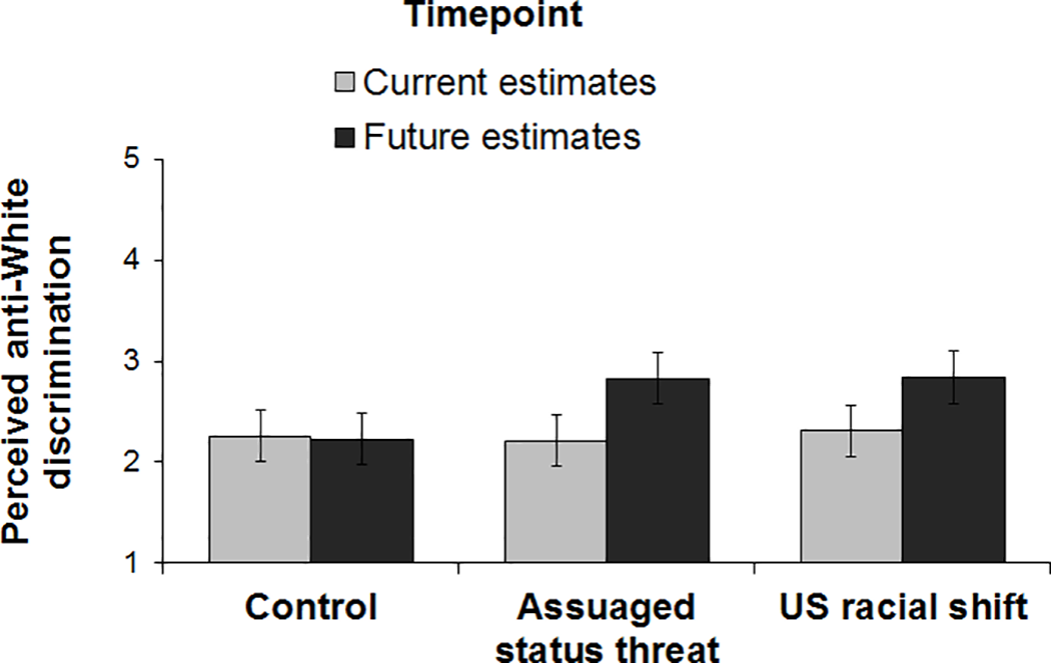
Perceived anti-White discrimination by experimental condition and timepoint (Study 2). Error bars represent within-subjects 95% confidence intervals [27].

A 3(condition: control, US racial shift, assuaged status threat) x 2(timepoint: current estimates, future estimates) mixed-design ANOVA on perceived anti-White discrimination revealed a main effect of timepoint, *F*(1, 222) = 12.58, *p* < .001, η_p_^2^ = 0.05, qualified by the experimental condition x timepoint interaction, *F*(2, 222) = 3.68, *p =* .027, η_p_^2^ = 0.03. As shown in Fig 2, similar to Study 1, participants in the control condition did *not* expect more anti-White discrimination in the future compared with current levels, *F*(1, 222) < 1, *p* = .884. Further replicating Study 1, participants in the US racial shift condition expected more anti-White discrimination in the future compared with current levels, *F*(1, 222) = 8.26, *p* = .004, η_p_^2^ = 0.04, as did participants in the assuaged status threat condition, *F*(1, 222) = 11.68, *p* = .001, η_p_^2^ = 0.05. Again, a contrast test (contrast codes: 2–1–1) revealed that, controlling for perceptions of current anti-White discrimination, participants in the control condition estimated less future anti-White discrimination than participants in either of the conditions in which the racial shift was salient, *F*(1, 221) = 7.55, *p* = .007, η_p_^2^ = 0.03.

Recall that participants also indicated how much Whites will face discrimination in the future in a number of domains (e.g., political influence, hiring decisions). Exploratory factor analysis using the principal axis method of factor extraction with oblique rotation revealed 2 factors (see Table 2 for factor loadings of all items) that correspond to a) the workplace and education (college admissions, scholarships, and hiring decisions; α = .85) and b) a variety of other, largely interpersonal and cultural domains (e.g., interpersonal interactions, dating, cultural expression, free speech; α = .91).

To examine whether Whites’ perceptions of increasing anti-White discrimination in response to the racial shift information were driven by expectations about facing more of a particular type of discrimination, we conducted a 3(condition: control, US racial shift, assuaged threat) x 2(type of discrimination: employment/education, interpersonal) mixed-design ANOVA on these expectations for *future* anti-White discrimination. Results revealed main effects of type of discrimination, *F*(1, 220) = 206.63, *p* < .001, η_p_^2^ = 0.48, and experimental condition, *F*(2, 220) = 8.00, *p* < .001, η_p_^2^ = 0.07, which were qualified by the interaction between the two factors, *F*(2, 220) = 4.33, *p =* .014, η_p_^2^ = 0.04. Analyses within each type of discrimination revealed an effect of experimental condition on anticipated future anti-White discrimination in employment/education, *F*(2, 220) = 8.23, *p <* .001, η_p_^2^ = 0.07. Participants in the control condition (*M* = 3.29, 95% CI[2.99, 3.60], *SD* = 2.18) anticipated less future anti-White discrimination in employment/education than both participants in the US racial shift condition (*M* = 4.80, 95% CI[4.50, 5.10], *SD* = 2.27), *F*(1, 220) = 16.19, *p* < .001, η_p_^2^ = 0.07 and those in the assuaged status threat condition (*M* = 4.23, 95% CI[3.93, 4.53], *SD* = 2.34), *F*(1, 220) = 6.32, *p* = .013, η_p_^2^ = 0.03, who did not reliably differ from one another, *F*(1, 220) = 2.37, *p* = .125, η_p_^2^ = 0.01. A similar, albeit weaker, pattern of results emerged for expectations about future anti-White discrimination in the interpersonal domains, *F*(2, 220) = 4.61, *p =* .011, η_p_^2^ = 0.04. Specifically, participants in the control condition (*M* = 1.88, 95% CI[1.57, 2.19], *SD* = 1.11) were less likely to expect future anti-White discrimination in the more interpersonal domains, compared with those in either the US racial shift (*M* = 2.50, 95% CI[2.20, 2.80], *SD* = 1.52), *F*(1, 220) = 6.43, *p* = .012, η_p_^2^ = 0.03, or assuaged status threat conditions (*M* = 2.55, 95% CI[2.25, 2.85], *SD* = 1.76), *F*(1, 220) = 7.48, *p* = .007, η_p_^2^ = 0.03. Again, these latter conditions did not differ from one another, *F*(1, 220) < 1, *p* = .847, η_p_^2^ = 0.00.

#### Perceived anti-racial minority discrimination

Replicating Study 1, analyses of the estimates of perceived discrimination against the different racial minority groups revealed only a reliable main effect of timepoint [Blacks: *F*(1, 222) = 238.89, *p* < .001, η_p_^2^ = 0.52; Latinos: *F*(1, 222) = 162.49, *p* < .001, η_p_^2^ = 0.42; Asian Americans: *F*(1, 222) = 79.20, *p* < .001, η_p_^2^ = 0.26; Native Americans: *F*(1, 222) = 95.74, *p* < .001, η_p_^2^ = 0.30]. Regardless of experimental condition, participants expected racial minorities to face less discrimination in the future than they do currently (see Table 1 for descriptive statistics).

### Discussion

Consistent with Study 1, making the increasing racial diversity of the US salient led White participants to anticipate experiencing more anti-White discrimination, compared with making geographic mobility (control information) salient. Study 2 revealed, further, that Whites expressed increased expectations about facing future discrimination, particularly in employment and educational domains, but also in a variety of interpersonal and more cultural domains. As in Study 1, the results of Study 2 suggest that participants’ increased expectations of anti-White discrimination do not solely stem from concerns about Whites losing their relatively high realistic (i.e., material) status position in society. Thus, in Studies 3 and 4 we sought to explore two alternative explanations for the effect of exposure to information about increasing US racial diversity on Whites’ perceptions of anti-White discrimination.

## Study 3

Given that participants in Study 2 reported heightened expectations that they will face discrimination in a variety of domains in an increasingly racially diverse nation, Study 3 tested whether providing participants with information that both individuals and institutions in the future will be less likely to consider racial category information when making decisions (i.e., will adopt a colorblind ideology), could reduce Whites’ expectations of increased future anti-White discrimination. Research finds that White Americans tend to prefer colorblind over more race-conscious strategies for navigating interracial interactions, as well as in employment and educational decision contexts [28]. Further, prior work suggests that Whites (particularly those who endorse the current racial status hierarchy) endorse colorblind ideology in response to threat in order to legitimize the racial status quo [29]. Thus, information ensuring that colorblind ideology will be prevalent in any future, more diverse US may also serve to reduce concerns that Whites may face racial bias. Study 3 examined this possibility.

### Method

#### Participants

Three hundred and seventy White participants (192 women, 177 men, 1 did not report gender, *M*_age_ = 38.71, *SD*_age_ = 13.00, median family household income reported as between $45,000 and $59,999) recruited from MTurk.com took part in the experiment for $0.50. Participants reported residing in 46 different US states. Data were collected in December 2015.

#### Procedure and materials

White participants provided informed consent and completed an initial set of demographic questions (e.g., race, gender). After, participants were randomly assigned to read an article reporting on a) the projected growth of the rate of geographic mobility in the US (*control* condition), b) the projected future US racial demographics (*US racial shift* condition), or c) the projected future US racial demographics with an additional paragraph stating that individuals and institutions will not consider racial category information in the future, especially for employment or educational decisions (*colorblind-future* condition; see S1 Appendix for full text). In the colorblind-future condition, for example, participants read that “because of the demographic changes, companies and universities will no longer use race in consideration of employment, admissions, or scholarship decisions.” Participants in the racial shift and colorblind-future condition subsequently completed a manipulation check item regarding the extent to which they perceived race was likely to be emphasized in the future (1 = *Emphasized* *less* *in the future*, 2 = *No change*, 3 = *Emphasized* *more* *in the future*). As in the previous experiments, all participants next indicated their perceptions that different racial groups currently face discrimination and will face discrimination in the future on the same scale (1 = *Not at all*, 10 = *Very much*) described previously. Last, participants completed additional demographic questions and were debriefed.

### Results

No participants were excluded from analyses (130 in the control condition, 120 in the US racial shift condition, and 120 in the colorblind-future condition).

#### Manipulation check

Analyses of the manipulation check revealed that participants in the US racial shift condition reported that people and society were more likely to emphasize race in the future (*M* = 1.94, 95% CI[1.78, 2.10], *SD* = 0.90) compared with participants in the colorblind-future condition (*M* = 1.27, 95% CI[1.15, 1.38], *SD* = 0.65), *t*(237) = 6.69, *p* < .001, *d* = 0.86.

#### Perceived anti-White discrimination

A 3(condition: control, US racial shift, colorblind-future) x 2(timepoint: current estimates, future estimates) mixed-design ANOVA on perceived anti-White discrimination revealed a main effect of timepoint, *F*(1, 365) = 28.72, *p* < .001, η_p_^2^ = 0.07, qualified by an experimental condition x timepoint interaction, *F*(2, 365) = 3.07, *p =* .048, η_p_^2^ = 0.02. Consistent with Studies 1 and 2, whereas participants in the control condition did not expect more anti-White discrimination in the future (*M* = 3.57, 95% CI[3.35, 3.78], *SD* = 2.83) vs. current levels (*M* = 3.38, 95% CI[3.16, 3.59], *SD* = 2.63), *F*(1, 365) = 1.43, *p* = .233, participants in the US racial shift condition expected that Whites would face more discrimination in the future (*M* = 4.04, 95% CI[3.82, 4.27], *SD* = 2.68) than they do currently (*M* = 3.32, 95% CI[3.10, 3.55], *SD* = 2.52), *F*(1, 365) = 19.85, *p* < .001, η_p_^2^ = 0.05. Interestingly, participants in the colorblind-future condition also perceived that Whites would face more discrimination in the future (*M* = 3.91, 95% CI[3.68, 4.13], *SD* = 2.75) than they do currently (*M* = 3.33, 95% CI[3.11, 3.56], *SD* = 2.48), *F*(1, 365) = 12.67, *p* < .001, η_p_^2^ = 0.03. As in Studies 1 and 2, a contrast test (contrast codes: 2–1–1) revealed that, controlling for perceptions of current anti-White discrimination, participants in the control condition estimated less future anti-White discrimination than participants in either of the conditions in which the racial shift was salient, *F*(1, 364) = 5.80, *p* = .016, η_p_^2^ = 0.02.

#### Perceived anti-racial minority discrimination

Analyses of the estimates of perceived discrimination against the different racial minority groups revealed a main effect of timepoint [Blacks: *F*(1, 365) = 170.35, *p* < .001, η_p_^2^ = 0.32; Latinos: *F*(1, 365) = 110.87, *p* < .001, η_p_^2^ = 0.23; Asian Americans: *F*(1, 363) = 56.59, *p* < .001, η_p_^2^ = 0.14; Native Americans: *F*(1, 363) = 88.14, *p* < .001, η_p_^2^ = 0.20]. Once again, participants perceived that racial minorities would face less discrimination in the future than they do currently (see Table 1 for descriptive statistics).

Unlike Studies 1 and 2, main effects of condition also emerged [Blacks, *F*(2, 365) = 9.70, *p* < .001, η_p_^2^ = 0.05; Latinos, *F*(2, 365) = 4.16, *p* = .016, η_p_^2^ = 0.02; Asian Americans, *F*(2, 363) = 5.97, *p* = .003, η_p_^2^ = 0.03; Native Americans, *F*(2, 363) = 4.90, *p* = .008, η_p_^2^ = 0.03]. These patterns of results reveal that participants in the colorblind-future condition expressed that racial minorities face less discrimination (both current levels and future levels) than did participants in the US racial shift condition [Blacks, *F*(1, 365) = 19.17, *p* < .001, η_p_^2^ = 0.05; Latinos, *F*(1, 365) = 7.44, *p* = .007, η_p_^2^ = 0.02; Asian Americans, *F*(1, 363) = 9.95, *p* = .002, η_p_^2^ = 0.03; Native Americans, *F*(1, 363) = 9.65, *p* = .002, η_p_^2^ = 0.03], or the control condition [for perceived anti-Black, *F*(1, 365) = 6.97, *p* = .009, η_p_^2^ = 0.02, and anti-Latino discrimination, *F*(1, 365) = 4.82, *p* = .029, η_p_^2^ = 0.01]. Control participants also expressed that Asian Americans faced or will face less discrimination than did participants in the US racial shift condition, *F*(1, 363) = 7.90, *p* = .005, η_p_^2^ = 0.02.

Further, interaction effects emerged for perceived discrimination against Blacks, *F*(2, 365) = 4.28, *p* = .015, η_p_^2^ = 0.02, and Latinos, *F*(2, 365) = 7.78, *p* < .001, η_p_^2^ = 0.04, such that participants who read that race would be emphasized less in the future (colorblind-future condition) had a steeper drop in perceived future discrimination against Blacks and Latinos (vs. the present), compared with participants in the control condition [Blacks, *F*(1, 365) = 8.53, *p* = .004, η_p_^2^ = 0.02; Latinos, *F*(1, 365) = 15.55, *p* < .001, η_p_^2^ = 0.04].

### Discussion

In sum, exposure to the increasing racial diversity of the US, compared with control information, leads Whites to expect that their group will face more discrimination in the future than they currently face. This result was not meaningfully attenuated by information that a future, more diverse US will be accompanied by a reduction in the salience and relevance of race (i.e., colorblind ideology), suggesting that concerns regarding race-conscious decision-making (in which Whites often presume they will be affected negatively) are not the root of Whites’ increased perceptions of anti-White discrimination in the future.

Further, the results for ratings of discrimination faced by racial minorities were unlike those of Studies 1 and 2, but consistent with work suggesting that colorblind ideology can promote the denial of anti-minority discrimination (see [30]). That is, participants for whom a future-colorblind “majority-minority” nation was salient reported the largest expected decline in anti-Black and anti-Latino discrimination, suggesting that whereas an alleged color-blind future may not decrease concerns about anti-White discrimination, it may reduce concerns about discrimination faced by some minorities.

## Study 4

Given that colorblind ideology did not influence Whites’ concerns about anti-White discrimination in an increasingly racially diverse United States, Study 4 tested one additional potential mechanism through which increasing racial diversity may influence Whites’ perceptions that they will face more discrimination in the future—namely, concerns that American society itself and what it means to be American will change. That is, rather than a message suggesting that race-conscious decision-making would be reduced in the future (as in Study 3), Study 4 explored whether information that the cultural identity of America would remain unchanged may reduce the observed effects for perceived anti-White discrimination. Indeed, previous research suggests that the more White Americans believe that the White population is shrinking relative to other racial groups, the more they report that ethnic/racial minorities should assimilate to White American cultural norms, customs, and values (see [11]); further, this relation is driven, at least in part, by concerns that Whites will lose their status as the “prototypical Americans.” Hence, it is possible that cultural “prototypicality threat” also increases Whites’ concerns about experiencing discrimination in a future, more diverse US. To test this possibility, White participants’ expectations regarding anti-White discrimination were examined after either reading about the shifting racial demographics of nation alone (US racial shift condition) or accompanied by information assuaging concerns that American culture and what it means to be American are unlikely to change (*assimilation-future* condition). Similar to the previous studies, both conditions were compared to a neutral control condition. If concerns about cultural change are at the root of Whites’ concerns about anti-White discrimination in the future, then participants in the assimilation-future condition should report expecting less anti-White discrimination in the future compared with participants in the US racial shift condition.

### Method

#### Participants

One hundred and fifty-four White participants (45 women, 109 men, *M*_age_ = 19.79, *SD*_age_ = 2.64, median family household income reported as between $90,000 and $99,999) at Ohio State University took part in the experiment for partial course credit or in exchange for $5. Data collection spanned January to April, 2016.

#### Procedure and materials

Participants provided informed consent and completed an initial set of demographic questions (e.g., race, gender). Participants read one filler article, followed by an article that either reported on a) the growth of the rate of geographic mobility in the US (control condition), b) the projected future US racial demographics (US racial shift condition), or c) the projected future US racial demographics with an additional paragraph stating that American society and what it means to be American will remain the same in the future (assimilation-future condition; see S1 Appendix for full text). In the assimilation-future condition, for example, participants read:

Scientists who examine the impact of demographic shifts in other contexts nearly unanimously agree that these assimilative processes will yield a more cohesive American society with unified values as minorities appreciate and conform to the mainstream culture. . . Because there will be no one racial group with over 50% of the population, people of all races will think of themselves as Americans first, and what it means to be American is likely to mirror what it means to be American today.

As a manipulation check, participants in the US racial shift and assimilation-future conditions were also asked to indicate their agreement that American society is likely to change drastically in the future (1 = *Strongly disagree*, 7 = *Strongly agree*). As in Studies 1–3, further, participants indicated how much different groups currently face discrimination followed by assessments about how much groups will face discrimination in the future (1 = *Not at all*, 10 = *Very much*). Last, participants completed additional demographic questions and were debriefed.

### Results and discussion

No participants were excluded from analyses (52 participants in the control condition, 52 in the US racial shift condition, 50 in the assimilation-future condition).

#### Manipulation check

Analyses of the manipulation check item revealed that participants in the US racial shift condition were more likely to report that American society is likely to change drastically in the future (*M* = 4.90, 95% CI[4.53, 5.28], *SD* = 1.39) compared with participants in the assimilation-future condition (*M* = 3.52, 95% CI[3.01, 4.03], *SD* = 1.84), *t*(100) = 4.29, *p* < .001, *d* = 0.85. Hence, the assimilation manipulation was successful in allaying concerns about American cultural change.

#### Perceived anti-White discrimination

**Fig 3:**
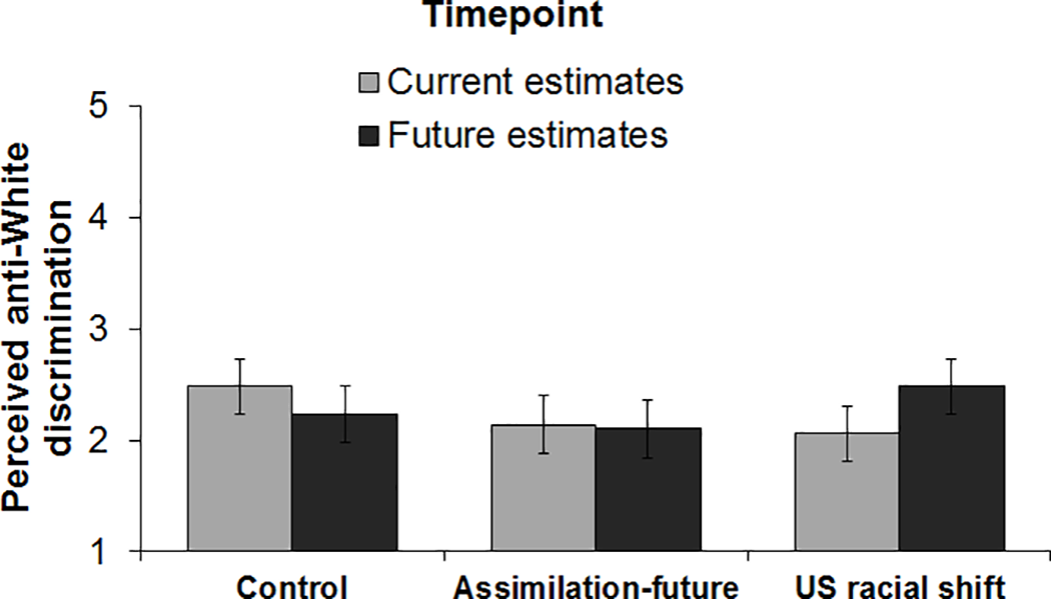
Perceived anti-White discrimination by experimental condition and timepoint (Study 4). Error bars represent within-subjects 95% confidence intervals [27].

To examine whether Whites’ expectations regarding racial discrimination may also be shaped by this assimilation information, we conducted a 3(condition: control, US racial shift, assimilation-future) x 2(timepoint: current estimates, future estimates) mixed-design ANOVA on perceived anti-White discrimination. Results revealed a reliable experimental condition x timepoint interaction, *F*(2, 151) = 3.69, *p* = .027, η_p_^2^ = 0.05. As depicted in Fig 3, participants in the control condition did not expect more anti-White discrimination in the future vs. present, *F*(1, 151) = 1.95, *p* = .165. Replicating the previous three studies, participants in the US racial shift condition expected that Whites would face more discrimination in the future than they do currently, *F*(1, 151) = 5.57, *p* = .020, η_p_^2^ = 0.04. Consistent with the idea that concerns about change in “American cultural norms” in a future “majority-minority” US are responsible for the elevation in perceptions of anti-White discrimination, participants in the assimilation-future condition did *not* expect that Whites would face more discrimination in the future than they currently face, *F*(1, 151) < 1, *p* = .827.

Further, a contrast test (contrast codes: 1–1 0) revealed that, controlling for perceptions of current anti-White discrimination, participants in the control condition estimated less future anti-White discrimination than participants in the US racial shift condition, *F*(1, 150) = 5.82, *p* = .017, η_p_^2^ = 0.04. However, controlling for perceptions of current anti-White discrimination, participants in the control condition did not reliably differ in their estimates of future anti-White discrimination compared with participants in the assimilation-future condition (contrast codes: 1 0–1), *F*(1, 150) < 1, *p* = .548.

#### Perceived anti-racial minority discrimination

Similar to Studies 1 and 2, analyses of the discrimination perceptions regarding the different racial minority groups revealed only the main effect of timepoint [Blacks: *F*(1, 151) = 120.77, *p* < .001, η_p_^2^ = 0.44; Latinos: *F*(1, 151) = 52.80, *p* < .001, η_p_^2^ = 0.26; Asian Americans: *F*(1, 151) = 60.24, *p* < .001, η_p_^2^ = 0.29; Native Americans: *F*(1, 151) = 75.93, *p* < .001, η_p_^2^ = 0.34]. Regardless of experimental condition, participants perceived that racial minority groups would face less discrimination in the future, compared to current levels (see Table 1 for descriptive statistics).

## General discussion

The results of four experiments (*N* = 931; see Table 3 for a summary of the key findings) offer consistent evidence that exposure to information about the changing US racial demographic landscape leads White Americans to perceive that their group will face increasing amounts of racial discrimination in a racially-diverse, “majority-minority” future. Further, we replicated prior research [10, 12] finding that information about the racial shift leads White Americans to express greater support for conservative policies, unless group status concerns are assuaged (Study 1). Concurrently, similar to the findings of past research [6], regardless of the information made salient (i.e., information about the racial demographic shift or control information), Whites estimated that racial minorities will face ever dwindling amounts of discrimination in the future. These results were observed among both student and online samples, lending confidence to the robustness of the effects. That is, the online sample (Study 3) was much more geographically diverse and generally reported lower household incomes, compared with the student samples (Studies 1, 2, and 4). Despite these demographic differences, consistent experimental effects were found across studies. *Post hoc* power analyses of each study indicated that we had 99% power to detect the predicted effects in all studies.

**Table 3:** Summary of findings (Studies 1–4).

Research question	Answer
Does information about increasing racial diversity increase Whites’ perceptions of discrimination faced by…	
White Americans	**Yes**
Racial minority groups	No
Do Whites expect discrimination to decline over time for…	
White Americans	No
Racial minority groups	**Yes**
Does reducing concerns that Whites will lose status alleviate the increases in perceived anti-White discrimination?	No
Does reducing concerns regarding race-conscious decision-making alleviate the increases in perceived anti-White discrimination?	No
Does reducing concerns that American society and culture are changing alleviate the increases in perceived anti-White discrimination?	**Yes**

Across studies, on average, participants reported that racial minorities (and, particularly, Blacks and Latinos) both currently face and will face more discrimination, compared with Whites. This is a somewhat different pattern of results than that revealed in previous work, which has shown Whites to perceive that they currently face more discrimination than Blacks [6]. One possible reason for this dissimilarity lies in the different time period comparisons participants made in this prior work (i.e., rating the extent of discrimination faced in each decade, from the 1950s to current levels) and in the present research (i.e., rating the extent of discrimination that groups currently face and will face in the future). However, despite Whites’ perceptions that minorities will generally face more discrimination than Whites do/will, no consistent effect of racial shift information was found for Whites’ estimates of anti-minority discrimination. This suggests that the racial shift information activates concerns about Whites’ (i.e., the ingroup’s) place in a future, more diverse US and that White Americans may perceive that in this future US, increased anti-White discrimination may not necessarily imply a matched amount of decreased anti-minority discrimination. These possibilities are beyond the scope of the present work, but may benefit from future research.

It is interesting that, unlike the outcomes of making the increasing racial diversity of the US salient found in recent research (e.g., increased racial bias, greater conservatism, more negative intergroup emotions; [9–10, 13]), the effects on Whites’ expectations of increased anti-White discrimination found in the present work were *not* primarily driven by increased concern about Whites’ group status in a future “majority-minority” US (Studies 1–2). Somewhat surprisingly, suggesting that race and race-conscious decision-making will no longer occur in a more diverse future US also did not reduce expectations regarding increasing anti-White discrimination (Study 3). Study 4, however, shed light on at least one potential mechanism underlying this effect. Specifically, if participants’ concerns that the increasing racial diversity of the US will fundamentally change dominant American culture and what it means to be American were assuaged (assimilation-future condition), their expectations that anti-White discrimination will rise were also eliminated. Consistent with recent work by Danbold and Huo (2015) [11], in other words, concerns that what it means to be “American” might change in ways that, presumably, are perceived to be detrimental to White Americans seem to fuel Whites’ concerns about facing anti-White discrimination in a future, more diverse United States.

Why might concerns regarding losing cultural dominance (but not concerns regarding losing status in terms of income and wealth) lead to perceptions that Whites will face more discrimination in the future? The results of Study 2, in which participants reported that Whites are likely to face increasing discrimination in both domains associated with wealth or material resources (e.g., education, employment) and in more interpersonal (e.g., dating) and cultural domains (e.g., free speech, cultural expression) may have hinted at this mechanism. It is possible, then, that cultural dominance is perceived to maintain Whites’ status across the host of domains, and thus, assuaging Whites’ concerns that they may lose cultural dominance was able to disrupt perceptions of increasing anti-White discrimination more generally. That is, the breadth of the perceived threat of facing discrimination across many domains may be reduced by information that suggests Whites will maintain their status across this wide variety of domains. Future research is still needed, however, to determine whether additional psychological mechanisms may also contribute to heightened perceptions of and concerns about anti-White discrimination.

Given the predominantly-male samples comprising the majority of studies in the present research, it is possible that the observed effects of the racial shift information on perceived anti-White discrimination may be primarily indicative of White men’s reactions to changing demographics. Study 3 had a gender-balanced sample and revealed no evidence that gender moderated the primary findings [*F*(2, 361) < 1, *p* = .786], which provides some confidence that the findings are indeed generalizable. Future research that specifically examines how White men and White women may (or may not) differ in their reactions to the changing demographics, however, is needed to fully explore this important question.

Future work is also needed to address the essential, but largely understudied, question of how the future “majority” reacts to the growing national ethnic diversity (for notable exceptions, see [31–32]). That is, the present work focuses on how Whites’ perceptions of discrimination are influenced by information that their group will become a smaller proportion of the total population; it is equally imperative for future work to examine how members of different racial minority groups’ expectations regarding discrimination may vary (or not) due to salient information regarding these changing demographics.

The implications of these findings are sobering. Most notably, the present work reveals the possibility that the growing racial diversity of the nation is likely to generate even more divergent perceptions held by Whites and members of racial minority groups regarding the extent to which Whites and racial minorities face discrimination than has been found previously [6, 33]. Specifically, the present findings suggest that the increasing racial diversity of the nation is likely to lead Whites to expect (and perceive) increasingly lower rates of discrimination against minorities and increasingly higher rates of anti-White discrimination. Perceptions that minorities face less discrimination currently compared with in previous decades has already been used to justify removing protections against that selfsame discrimination (e.g., [5]), arguably, to devastating effects for voter enfranchisement [34]. Moreover, perceptions that Whites face an increasing prevalence of discrimination have similarly contributed to recent Supreme Court decisions that have narrowed the scope of affirmative action in undergraduate admissions [35] and struck down even voluntary school desegregation efforts [36]. Such perceptions are also likely to increase the number of discrimination claims filed by White plaintiffs [37], which, in turn, will reinforce the perception that anti-White discrimination is increasing and, perhaps, that anti-racial minority discrimination is declining.

Despite these perceptions, discriminatory practices against racial minorities still occur with alarming regularity (e.g., [38]; for a recent review see, [39]). Indeed, in nearly every important domain of American life, including health, education, criminal justice, and wealth, substantial racial disparities (favoring Whites) continue to persist and discrimination has been found to contribute to these gaps [39–42]. The present findings reveal the underlying psychological processes through which the growing racial diversity of the nation may unwittingly make it increasingly difficult to address these troubling disparities and cultivate a nation that is both diverse and just.

## Supporting information

S1 Appendix Perceived discrimination measures and experimental materials. (DOCX)
