# Supplementary material for: Information about the US racial demographic shift triggers concerns about anti-White discrimination among the prospective White “minority”
Source: PLoS One. 2017 Sep 27;12(9):e0185389. doi: 10.1371/journal.pone.0185389 (PMC5617190; doi:10.1371/journal.pone.0185389)
Supplement: S1 Appendix — (DOCX) [file pone.0185389.s001.docx]

**S1 Appendix**

**Perceived discrimination measures**

**Current vs. future estimates (Studies 1-4)**

*Instructions*: Please indicate how much you think different groups ***are currently*** facing discrimination in the United States.

*Scale*: 1 = Not at all, 10 = Very much

*Groups*: White Americans, Black Americans, Hispanics/Latinos, Asian Americans, Native Americans

*Instructions*: Please indicate how much you think different groups ***will face*** discrimination in the United States ***in the future***.

*Scale*: 1 = Not at all, 10 = Very much

*Groups*: White Americans, Black Americans, Hispanics/Latinos, Asian Americans, Native Americans

**Specific domains (Study 2)**

*Instructions*: For the following domains, please indicate how much you think White Americans may face discrimination in the United States in the future. That is, how likely are Whites to face discrimination in...

*Scale*: 1 = Not at all, 10 = Very much

*Domains*: Scholarships, College admission, Hiring decisions, Interpersonal interactions

Interactions with police, Housing, Dating, Free speech, Expressing their culture/traditions, Political influence

**Experimental materials**

The articles utilized to prime the US racial shift and control information can be found in the Supplemental Materials of Craig & Richeson (2014b; S3): <http://pss.sagepub.com/content/25/6/1189>

**Assuaged threat paragraph (presented after the US racial shift information for participants in the assuaged-threat condition of Studies 1 & 2)**

Despite the shift in the demographic make-up, the relative societal status of different racial groups is likely to remain steady. Largely due to continuing differences in educational attainment, White Americans are likely to remain the majority in powerful corporate and political positions. White Americans are expected to continue to have higher average incomes and wealth compared to members of other racial groups. Overall, despite the numerical shift, racial groups' relative positions in society are likely to remain the same as they are now.

**Colorblind paragraph (presented after the US racial shift information for participants in the colorblind-future condition of Study 3)**

Scientists who examine the impact of demographic shifts in other contexts nearly unanimously agree that a majority-minority society will be less likely to emphasize racial group differences. Because there will be no one racial group with over 50% of the population, people of all races will think of themselves as Americans first. Recent survey data supports this prediction. Further, because of the demographic changes, companies and universities will no longer use race in consideration of employment, admissions, or scholarship decisions. Some companies and universities have already begun to follow color-blind admissions and hiring policies with many more planning to do so in the coming decades. Overall, the demographic shift will likely lead to less of an emphasis on race from both individuals and institutions.

**Assimilation paragraph (presented after the US racial shift information for participants in the assimilation-future condition of Study 4)**

Despite the shift in the demographic make-up, American society is likely to remain relatively unchanged. Examining data spanning the past 100 years, demographers have found that immigrants and racial minorities are increasingly and overwhelmingly assimilating to the American way of life. Scientists who examine the impact of demographic shifts in other contexts nearly unanimously agree that these assimilative processes will yield a more cohesive American society with unified values as minorities appreciate and conform to the mainstream culture. These longstanding processes of assimilation reveal that the predominantly White mainstream culture will be maintained for the foreseeable future. Because there will be no one racial group with over 50% of the population, people of all races will think of themselves as Americans first, and what it means to be American is likely to mirror what it means to be American today.
